# The Clinical Obesity Maintenance Model: An Integration of Psychological Constructs including Mood, Emotional Regulation, Disordered Overeating, Habitual Cluster Behaviours, Health Literacy and Cognitive Function

**DOI:** 10.1155/2013/240128

**Published:** 2013-02-14

**Authors:** Jayanthi Raman, Evelyn Smith, Phillipa Hay

**Affiliations:** ^1^School of Medicine, University of Western Sydney, Locked Bag 1747, Penrith, NSW 2751, Australia; ^2^School of Psychiatry, University of New South Wales, 34 Botany Street, Randwick, NSW 2031, Australia

## Abstract

Psychological distress and deficits in executive functioning are likely to be important barriers to effective weight loss maintenance. The purpose of this paper is twofold. First, in the light of recent evidence in the fields of neuropsychology and obesity, particularly on the deficits in the executive function in overweight and obese individuals, a conceptual and theoretical framework of obesity maintenance is introduced by way of a clinical obesity maintenance model (COMM). It is argued that psychological variables, that of habitual cluster Behaviors, emotional dysregulation, mood, and health literacy, interact with executive functioning and impact on the overeating/binge eating behaviors of obese individuals. Second, cognizant of this model, it is argued that the focus of obesity management should be extended to include a broader range of maintaining mechanisms, including but not limited to cognitive deficits. Finally, a discussion on potential future directions in research and practice using the COMM is provided.

## 1. Introduction

Obesity, an excessive accumulation of fat in an individual, is an important public health issue and contributes to serious health problems and extensive economic costs worldwide [1]. A common measure of adult obesity is body mass index, defined as a person's weight (in kilograms) divided by the square of his or her height (in meters), above 30 [2]. Although weight loss is associated with clear health benefits, the prevention of weight regain has remained a challenge. Even with the help of professionals and extended behavioral treatments, weight regain typically occurs when professional contact ends [3]. By 3–5 years posttreatment, about 85% of the patients would have regained weight or even exceeded their pretreatment weight [4].

Recommendations for obese individuals include losing and maintaining a loss of 5–10% of body weight in order to reduce the risk of developing chronic medical conditions [5]. Although a 10% weight loss may not return an obese to a nonobese state, the positive health benefits of a 10% weight loss are well documented [6]. Some studies show correlations between long-term weight loss and continued consumption of low-fat diets, increased physical activity, self-monitoring weight, and maintaining a consistent eating pattern across weekdays and weekends [7–9]. It is unclear though how some formerly obese individuals are able to persevere with healthy behaviors when the majority find it difficult to do so [10].

A randomized controlled trial [11] explicitly designed to minimize posttreatment weight regain investigated the results of cognitive-behavioral therapy (CBT) on one hundred and fifty female obese participants but found little evidence to support a cognitive-behavioral approach to the treatment of obesity [12]. The literature therefore has been sparse on the psychological predictors of obesity maintenance and few baseline characteristics have been consistently predictive of subsequent outcomes [13]. 

A frequent criticism of obesity research is also the lack of a sound theoretical background, and researchers have stated the need for theory-based formulations in obesity research [14–16]. In the absence of robust empirical evidence, current interventions for obesity have largely relied on anecdotal clinical evidence [17, 18]. Hence an informed understanding of how to best treat this group of patients remains elusive.

This paper reviews and discusses extant literature on the psychological and neuropsychological aspects of obesity-management and aims to elucidate important influences on obesity maintenance. In particular, past research has overlooked the role of executive function (higher-level cognitive processes) interacting with obesity maintaining mechanisms. Current studies have indicated a negative association between obesity and cognition, especially in the area of executive function [19], irrespective of the presence of binge eating [20]. Recent findings in the field of cognitive neuropsychology and obesity are thus discussed. Additional psychological barriers to successful weight maintenance in the obese are reviewed. A novel theoretical framework by way of a clinical obesity maintenance model (COMM) is proposed. In the final sections of the paper, the broader relevance of the COMM is discussed.

## 2. Theoretical Rationale for the Constructs of the COMM

**Figure 1:**
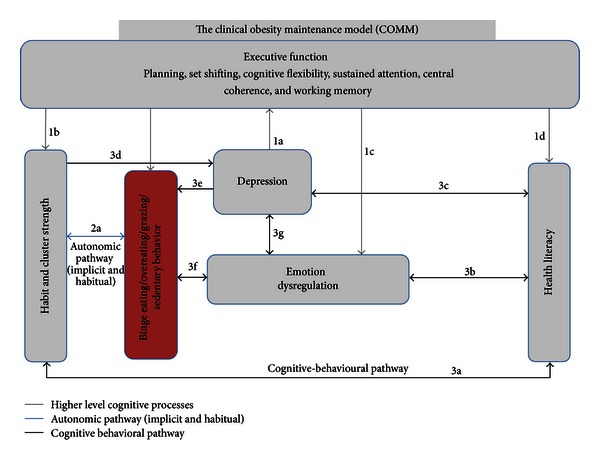
Hypothesized model of the Clinical Obesity Maintenance Model (COMM). *Note*. The letters indicate direct pathways between variables.

A review of recent findings in the field of behavioral and cognitive psychology is synthesized in this section to provide the rationale for the proposed Clinical Obesity Maintenance Model (COMM). It is proposed that a network of interrelated psychological mechanisms accounts for the maintenance of obesity with or without an accompanying a binge eating disorder. This Clinical Obesity Maintenance Model is illustrated in schematic form in Figure 1.

### 2.1. Executive Function in Obesity

The first of the obesity maintaining mechanisms concerns the influence of executive function. Executive function has been described as encompassing a range of cognitive processes “facilitating initiation, planning, regulation, inhibition, sequencing, and attainment of complex goal-oriented behavior and thought” [21], all of which may impact on eating behavior. Recent studies have indicated a negative association between obesity and cognition, especially in the area of executive function [19], irrespective of the presence of binge eating [20]. A systematic review of 34 studies by Smith et al. [19] found a consistent association between obesity and low scores on executive function in children, adolescents, and adults, even after controlling for socioeconomic status or medical comorbidities [19]. 

For example, studies have shown that obese participants show a pronounced impairment in decision making in the Iowa gambling task (IGT), a neuropsychological task of executive function designed to simulate real-life decision making and learning in terms of reward and punishment [22, 23]. Obese individuals perform similarly on this task compared to those with anorexia nervosa [24] and to those with orbitofrontal dysfunction [25] and worse than those who are dependent on substance use [26]. 

In addition, similar to individuals with anorexia nervosa [27], obese individuals have impaired central coherence (a detail-focused processing style, defined by not being able to see the “big picture”), but in contrast to those with anorexia nervosa they are very impulsive [28]. Finally, obesity has been shown to be associated with poorer performance on tests of global cognitive function and memory (e.g., [28]). The mechanism(s) by which obesity results in cognitive impairment however are uncertain. Postulated mechanisms include the effects of hyperglycemia, hyperinsulinemia, and vascular damage to the central nervous system [29]. Obesity could also impact on brain functioning by inflammatory mechanisms [30]. 

 The relationship between obesity and cognitive function is likely to be bidirectional. Two recent studies have shown that low cognitive performance predicts increased levels of adiposity [31, 32]. However, as documented by Smith et al., [19] no trials have yet investigated the effects of remediation of cognitive deficits on weight loss and/or weight loss maintenance. 

### 2.2. Habitual Cluster Behaviors

Empirical evidence indicates that when behaviors are underpinned by habits, they are autonomically enacted by the presence of cues or internal drive states [33, 34]. When aiming at changing these behaviors, the intentions need to be powerful enough to override the existing habits [33]. Habitual behaviors are maintained because of (a) mental efficiency, that is, speed and ease with which past patterns of behavior can be initiated and executed, (b) automaticity, that is, unawareness of performing the behavior, (c) susceptibility to cues by prevailing environmental events, and (d) these patterns are consistent with short-term goals [33, 35]. 

The ongoing persistence required to shift well-established behavior patterns is exemplified in the field for weight control [33]. A systematic review and meta-analysis by Gardner et al. [36] showed that the strongest habits were reported in relation to physical inactivity. Eight of nine studies in this meta-analysis provided evidence that habits moderated the intention-behavior relationship such that the impact of intention on behavior diminished as habit strength increased [36]. 

Few studies of behavior change in the field of obesity have attempted to formally incorporate the role of habit. In a field study of healthy eating, on a sample of 320 university students, Verplanken and Faes [37] confirmed the deleterious effects of counterintentional habits (e.g., eating fatty foods). This study demonstrated a negative relationship between counterintentional habits and performance of health behavior. Recent studies have begun to employ a validated measure of habit [38] in both dietary behaviors and physical activity [39]. Results have indicated that habit strength considerably adds to the extent of variance in behavior across various age groups (e.g., fruit consumption [40], saturated fat consumption [40], adolescent screen-viewing behavior associated with consumption of sugar-sweetened beverages [41], and physical activity [42]). One reason why long-term behavior change may be difficult for the obese population is that the behaviors that individuals want to change are relatively habitual, such as poor dietary habits, sedentary behaviors, and environmental behaviors.

*Clustering* refers to the cooccurrence of life-style-related risk factors that can be expected based on the prevalence of the separate behaviors [43]. Among adults, smoking and alcohol consumption have been shown to correlate with consumption of fewer fruits and vegetables and more fat [44, 45]. Smoking, excessive alcohol use, an unhealthy diet, and physical inactivity are the “big four” modifiable causes of obesity [46]. Empirical evidence indicates that these unhealthy behavior clusters tend to occur in combination within individuals [47–49] and among those who are obese [50]. 

Sedentary activity, in particular TV viewing, has been associated with unhealthy eating practices and may in part explain the relationship between sedentary behavior and obesity [51, 52]. Kushner and Choi [53] investigated the prevalence of unhealthy eating, exercise, and coping pattern traits among a large sample comprising of 18–65-years-old, 446,608 healthy-weight, overweight, and obese adults. This study found that the prevalence of these patterns rose with increasing BMI and that unhealthy lifestyle patterns in diet, exercise, and coping were highly prevalent among the overweight and obese population. The authors concluded that pattern recognition represents a new method to analyze the cluster of behaviors, attitudes, and traits seen among this population [53]. A better understanding about the prevalence, co-occurrence, and correlates of behaviors of obese individuals is needed to inform the design of interventions for weight management [54]. Presently, there is no empirical evidence regarding the maladaptive behavioral clusters in the obese that are extended with habit strength.

### 2.3. Emotional Dysregulation in Obesity

Emotional regulation requires a person to be aware of internal experiences (neutral, negative, and positive internal emotional states), identify the emotion, and effectively cope with or tolerate the emotion [55, 56]. Emotional arousal typically depresses one's appetite through a reduction in gastric hunger contractions. In contrast, obese individuals demonstrate a dysregulated physiological response to intense emotion by tending to increase their food intake during periods of emotional arousal and/or stress, a response known as emotional eating [57–60]. 

 Negative mood states are triggers for overeating in obese/overweight samples and overeating/binge eating may function as strategies to escape, avoid, or minimize negative affect [61–64]. For example, a study by Jansen et al. [65], on overweight/obese and normal weight participants, clustered into high- and low-negative-affect subtypes, found that negative mood induction and food exposure elicited overeating in the overweight/obese high negative affect subtype when compared to overweight/obese low negative affect subtype and normal-weight controls. Thus, while negative affect alone does not contribute to overeating and obesity, there is certainly a subsample of obese individuals who overeat when they are feeling low. Although there is substantial empirical support for a direct link between emotional dysregulation and binge eating disorder (see [66]), at present, there is little understanding of the maladaptive ways in which obese individuals cope with deficits in emotion regulation.

### 2.4. Depression in the Obese

A major depressive episode is characterized by periods of 2 weeks or more of depressed mood and/or anhedonia, in conjunction with other symptoms, including significant disturbance in appetite, sleep, psychomotor retardation or agitation, loss of energy, concentration problems, feelings of worthlessness or guilt, and/or suicidal ideation or intent [67]. An association between depression and obesity has been observed in both epidemiological and clinical studies [68, 69] with recent meta-analyses on both cross-sectional and prospective studies giving evidence for such an association [70, 71]. A study by Petry et al. [72] found that the prevalence of depression increased with increasing degrees of obesity. Differences were apparent for past-year depression as well with prevalence rates of 11.4% for class 1/II obesity and 16.2% for class III obesity [73]. Among weight-loss-seeking obese individuals, prevalence of depression is even higher, with 19% to 50% reporting a lifetime history of depression [74, 75]. 

A meta-analysis by Luppino et al. [71] found a longitudinal, reciprocal link between depression and obesity. Obesity was found to increase the risk of developing depression and depression was found to be predictive of obesity. A study by Renn et al., [75] also found bidirectional associations between depression and obesity: obese individuals had a 55% increased risk of developing depression over time, whereas depressed persons had a 58% increased risk of becoming obese. This study found “a dose-response gradient” in that the association between depression and obesity was stronger than the association between depression and overweight [75]. Although the causes for the obesity-depression link are uncertain, several possible theories have been discussed including the following: (a) obesity exacerbates inflammatory markers and these markers in turn have a role in the development and maintenance of depression [70]; (b) obesity contributes to body dissatisfaction and low self-esteem, putting the overweight individual at risk for depression, especially in treatment-seeking and morbidly obese individuals [70, 75]; (c) depression may increase weight by through central neuroendocrine effects either primary or secondary to antidepressant use [70, 76, 77].

The exact nature of the relationship between depression and obesity maintenance remains unclear, perhaps because clinical depression is a common exclusion criterion in weight-loss intervention trials [78, 79]. It is important to further elucidate how these two major health problems interact, so that prevention and treatment strategies are improved [70]. 

### 2.5. Health Literacy

One other construct that has been included in the proposed COMM is that of health literacy. “In order to understand and respond to the difficulties presented by a chronic condition, individuals construct their own common-sense model of their condition comprising of their belief sets and attitudes” [80, 81]. In the area of mental health, researchers [82, 83] have argued that poor mental health literacy, that is, poor awareness and understanding of the nature and treatment of health problems, is a major factor in the individual, social, and economic burden of mental health problems. Poor health literacy also includes attitudes and beliefs likely to be conducive to stigmatization of and discrimination against a condition or illness [84, 85]. In the field of obesity, it has been postulated that interventions that include health literacy will produce greater effects than standalone behavioral approaches (e.g., [86]). 

The health literacy construct in the proposed model will add to the multidimensional nature of the obesity experience by including (a) risk awareness/consequences of binge eating/overeating, (b) perceived personal control over condition/treatment, (c) perception of barriers to recovery and maintenance of successful weight management, and (d) other obesity-related beliefs and attitudes. Health literacy is addressed in part in behavior weight loss treatments with psychoeducation. It is not however understood how much this changes attitudes and beliefs of people with weight disorder and the efficacy of more specific health literacy interventions in obesity is unknown.

## 3. The Clinical Obesity Maintenance Model

In line with the above discussion, the proposed Clinical Obesity Maintenance Model (COMM: Figure 1) draws upon previous research and includes the following components from the cognitive and behavioral principles that are hypothesized to regulate weight management behaviors: (a) executive function, (b) habitual cluster behaviors, (c) emotion dysregulation, (d) depression, and (e) health literacy. 

It is reasoned that by delineating the role of emotional processes, the strength of habitual cluster behaviors, and executive functioning, the multidimensional nature of obesity maintaining behaviors is elucidated more clearly to inform scientific literature and clinical practice.

The suggested constructs may not necessarily operate simultaneously, nor may they be active in every case. Their partial independence may in part account for the heterogeneity and complexity that is found in the maintenance of obesity. It is likely, however, that important interactions among predictors exist. For example (see 1a in Figure 1), there is an ample evidence that depressive disorders are associated with deficits in attentional functions, executive control, lowered cognitive flexibility [87], verbal learning, memory [88], and interpretation biases [89–92]. Due to the impaired cognitive processes often associated with depression, obese individuals with depression may be less able to adhere to the recommended dietary and physical activities [69, 93]. A converse relationship may also apply (see 3d, 3e in Figure 1). Habit strength may mediate the relationship between depression and obesity maintaining behaviors [94] through cognitive means. For example, studies have shown that obese individuals who regain weight show a perceived lack of personal control over their weight management and a dichotomous thinking style, that is, an all or nothing thinking style about their eating behaviors [95, 96]. Hence when they are unable to resist strong habits, they may tend towards depressogenic thought patterns.

In addition, the COMM proposes that poor health literacy (see 3c in Figure 1) is a logical prerequisite for the development and maintenance of depressogenic beliefs and attitudes [83] about the obesity condition. Studies that have examined the impact of health literacy on the obese have generally relied on the quality of life as an outcome measure (e.g., [97, 98]). Little research has been done on the link between poor health literacy and depression with regard to obesity maintaining behaviors. The COMM aims to bridge this gap by proposing a cognitive-behavioral pathway (see 3c in Figure 1) in which poor health literacy contributes to the development of information-processing biases that increase the risk of depression in obese individuals. 

Research has shown that when cognitive resources become limited (i.e., deficits in executive function), individuals are inclined to make heuristic-based choices [99]. This is reflected in obesity maintaining dietary habits (see 1b in Figure 1). Given the habitual nature of eating [100] and the rapidity with which people make eating decisions [101], they are likely to be the consequence of automatic responses to contextual food cues, many of which lead to increased caloric consumption and poor dietary choices [99, 101].

In addition, emotion regulation has been described as a continuous, dynamic system responsive to all emotional experience, consisting of both autonomic and controlled processes [102, 103], perhaps mediated by executive function (see 1c in Figure 1). As it relates to obesity, problems in attentional and inhibitory control have been associated with binge eating and eating pathology in adults [104]. Although deficits in attentional control in obesity may contribute to behavioral impulsivity related to eating, emotion regulation as a self-regulatory process may control the affective processes that relate to eating (see 3f in Figure 1). Thus when obese and depressed individuals have a poor repertoire of emotion regulation strategies (e.g., deficits in inhibitory control), they may be unable to withhold inappropriate prepotent responses that have accumulated habit strength (see 3f in Figure 1). Furthermore, there may be a bidirectional association between depression and emotional dysregulation (see 3g in Figure 1). Evidence indicates that depression is characterized not only by poor mood levels (i.e., low positive affect and high negative affect [105, 106], but also by emotion dysregulation (for a meta-analysis, see [107, 108]). 

## 4. Conclusion

According to Anderson et al. [109] psychologically oriented research in the context of obesity maintenance is noteworthy for its tendency to focus on a limited range of psychological constructs, its limited conceptualization of psychological difficulties, and for its inconsistent findings. Researchers (e.g., [95, 110, 111]) have reiterated the need to examine the behavioral and psychological mechanisms that underpin longer-term weight management. As Cooper et al. [11] stated, the cognitive-behavioral model of obesity as a standalone model does not fully explain nor address the mechanisms that contribute to weight regain. The Clinical Obesity Maintenance Model (COMM) contrasts with earlier models focused on the cognitive and behavioral pathways maintaining obesity, by taking into account additional salient constructs such as habitual cluster behaviors, emotion dysregulation, health literacy, and executive deficiencies. Overlooking important cognitive and psychological barriers in the weight loss maintenance phase may perpetuate a sense of failure in the obese people and further undermine the low sense of self-esteem and self-efficacy of these individuals [112].

The COMM is presented as a psychological model and does not aim to include all the medical and sociocultural features associated with obesity. This model contains psychological features on the basis of extant empirical literature implicating a strong association between the proposed constructs. It is suggested that an integrative, multiprong approach may prove remedial in the assessment and treatment of obesity maintenance. For example, obesity-specific health literacy may improve the attitudes and beliefs regarding their weight management. Behavioral therapy can be specially formulated to target habitual cluster behaviors. Teaching emotion regulation strategies [55, 56] that affect weight management behaviors and employing cognitive-behavioral therapy for depression [113] may enable better coping skills with negative emotion and improve mood levels. As proposed by Smith et al. [19], executive function deficits may be addressed by cognitive remediation therapy. The COMM is planned to be further evaluated in empirical studies which include structural equation modeling and randomized controlled trial testing on interventions based on aspects of the model.
